# Resources to implement general population mobile chest x-ray screening for tuberculosis in Uganda: A trial-based costing analysis

**DOI:** 10.1371/journal.pgph.0005950

**Published:** 2026-09-10

**Authors:** Tyler Scott Johnson, David Isooba, Susan Birabwa, Juliet Kakeeto, Kenneth Ndyabayunga, Deborah Nantale, Ivan Mugabi, Violet Nakiiza, Annet Nalutaaya, Peter James Kitonsa, Emily A. Kendall, Achilles Katamba, David W. Dowdy

**Affiliations:** 1 Department of International Health, Johns Hopkins Bloomberg School of Public Health, Baltimore, Maryland, United States of America; 2 Uganda TB Implementation Research Consortium, Walimu, Kampala, Uganda; 3 Division of Infectious Diseases, Johns Hopkins University School of Medicine, Baltimore, Maryland, United States of America; 4 Clinical Epidemiology and Biostatics Unit, Department of Internal Medicine, Makerere University College of Health Sciences, Kampala, Uganda; 5 Department of Epidemiology, Johns Hopkins Bloomberg School of Public Health, Baltimore, Maryland, United States of America; University College of London, UNITED KINGDOM OF GREAT BRITAIN AND NORTHERN IRELAND

## Abstract

Mobile chest X-ray (CXR) screening interventions are recommended for tuberculosis (TB) screening in high-burden settings, but implementation costs are variable and underreported. Using time-driven activity-based costing, we evaluated the costs of implementing general-population mobile CXR screening in peri-urban Uganda. We conducted a micro-costing analysis of mobile CXR screening using data from an ongoing randomized crossover trial. We adopted a healthcare payer perspective, categorizing costs into equipment, personnel, space, implementation, and consumables. We calculated per-participant costs for each step in the TB screening cascade, as well as total screening and diagnostic costs per positive microbiological result (Xpert Ultra). Sensitivity analyses evaluated the cost implications of varying the screening volume or radiographic risk score thresholds. Among 90,668 age-eligible participants completing CXR screening, 14,630 sputum specimens were collected for Xpert testing (16.1%), including 1,676 from participants who submitted sputum without completing CXR. Overall, 511 participants had valid Xpert-positive results (3.5%), corresponding to 5.6 Xpert-positive results per 1,000 CXRs performed. The estimated cost was $4.41 per person screened and $1,299 per Xpert-positive result (95% uncertainty interval: $1,134-$1,488). Screening and diagnostic costs accounted for 64% and 36% of the cost per Xpert-positive result, respectively. Daily screening volume strongly influenced the cost per positive Xpert result, whereas less stringent X-ray thresholds increased the number of TB cases detected with minimal impact on average cost per positive Xpert. Mobile CXR screening interventions for TB require substantial investment in resources. Program planners can optimize efficiency by targeting high-burden populations, increasing participant volume, and optimizing X-ray risk cutoffs for follow-up diagnostic testing. Our findings highlight the value of context-specific cost data for guiding decisions on scaling up TB screening interventions.

## Introduction

Tuberculosis (TB) is the leading cause of death worldwide from a single infectious agent, despite the existence of several effective screening and treatment interventions [1]. Data from prevalence surveys indicate that approximately half of individuals with infectious TB are asymptomatic [2]. The World Health Organization (WHO) End TB Strategy highlights gaps in the detection of TB as key barriers that must be addressed to reduce global TB morbidity and mortality [3].

Active case finding (ACF) seeks to proactively identify individuals with TB before they present with symptoms [4]. Previous studies have specifically demonstrated the effectiveness of ACF using chest x-ray (CXR) as a TB screening tool [5–8]. Computer-aided detection (CAD) uses software to analyze chest radiographs for TB-related abnormalities, providing AI-generated scores for screening or triage [9]. Other studies have established the diagnostic accuracy of CAD models, but there is a need for implementation data on CAD-assisted ACF for TB in high-burden settings [10,11].

Resource constraints make accurate cost estimates essential for National TB Programs (NTPs) and their funders to allocate resources efficiently, effectively, and equitably [12]. Interest in community-based CXR is growing among NTPs and implementing partners [13,14]. However, the substantial human resource and infrastructure requirements required to support such screening campaigns represent a considerable barrier to scale-up [10,15]. Understanding the detailed costs of CXR-based screening for TB can therefore help to inform evidence-based resource allocation decisions.

Using a micro-costing approach, we sought to [1] enumerate the costs of implementing mobile CXR screening for TB in Uganda in communities and health facilities; [2] calculate the costs per individual undergoing initial screening as well as follow-up diagnostic testing; and [3] estimate the cost per positive test result for TB disease (Xpert Ultra), including how these costs vary as a function of key operational variables.

## Methods

### Ethics statement

The Ethics Review Committees of the Makerere University School of Public Health (Study reference number: SPH-2021–181) and Johns Hopkins School of Medicine (IRB protocol number: IRB00300939) approved the study. Participants provided informed verbal consent, which was documented by trained research staff on study tablets. For participants aged 15–17, consent was obtained from a parent/guardian and assent was obtained from the adolescent participant, as approved by the Institutional Review Board.

We performed micro-costing analyses as part of an ongoing cluster-randomized crossover trial (CHASE-TB; ClinicalTrials.gov: NCT05285202) conducted in eight health-facility catchment-area clusters within 200 km of Kampala, Uganda. The trial was designed to investigate the effectiveness and implementation of two strategies for implementing mobile CXR screening for TB: a “community-based” strategy that screened people in public locations identified by local clinic staff as having the highest likely prevalence of undiagnosed TB, and a “facility-adjacent” strategy that screened people at a fixed location adjacent to the catchment area’s primary health facility (a district hospital or other public health facility of similar scale). Clusters were randomized to alternating strategies for four months at a time, interspersed with four-month “washout” periods of no screening. Consenting participants ≥15 years of age were offered CXR, with analysis by CAD (qXR, Qure.ai); and participants whose qXR scores were above a pre-specified threshold (initially 0.5, but subsequently lowered to 0.1 on scientific grounds) were offered Xpert® Ultra testing (Cepheid, Sunnyvale, CA, USA). For a specified period, eligible participants were offered tuberculin skin testing (TST) to assess TB infection (TBI).

### Screening data

To estimate the number of participants per screening day (screening volume) and the proportion of participants completing each step of the diagnostic cascade, we evaluated data from participants screened from November 26, 2022, to April 17, 2025 (n = 90,717, 45,900 at community-based sites and 44,817 at facility-adjacent sites), during which a qXR threshold of 0.1 was used to select participants for sputum Xpert testing.

### Time measurements

We derived staff time estimates from observations of staff and participant time-and-motion (TAM) data collected from July 2023 to April 2024 to generate a process map outlining the staff time required to facilitate each screening step [16]. We separated screening steps into those required for initial screening for all participants and those completed by a subset of participants undergoing additional diagnostic testing for active TB or TBI. We split counseling time into follow-up counseling for all participants receiving X-rays and counseling specifically for participants providing sputum samples for Xpert testing. We did not account for time spent calling participants after hours or visiting participants outside of the screening site.

We used participant TAM data to allocate costs to each screening step in proportion to the total time spent on that step across all participants screened each day. We do not examine costs associated with TST testing, but we have maintained these time estimates in the process map because they are relevant for enumerating the time spent across all screening activities.

### Resource costs

We adopted a healthcare payer perspective, focusing on health system costs required to support implementation of the screening intervention (i.e., excluding research costs not required for programmatic implementation). Costs were reported in 2024 US dollars. We categorized intervention costs at each site into the following resource groups: equipment, personnel, physical space, implementation activities, and consumables. We used empirical study data to estimate costs for all resources, except for equipment and consumable costs per Xpert test, which were previously published in a similar Ugandan context [17].

A priori, we defined costs as either fixed (independent of screening volume) or variable (proportional to screening volume). To estimate costs associated with fixed resources (equipment, personnel, space, and implementation), we first calculated the capacity cost rate (CCR) for each resource, defined as the cost of capacity-supplying resources divided by the practical capacity of that resource (i.e., the time that the resource was available to support the screening intervention or the volume of participants screened with the resource) [18]. Equations for each CCR calculation are provided in S1 Appendix.


$$\begin{array}{c} CCR=\;\frac{Resource\;cost\;(\$)}{Practical\;capacity\;(minutes,\;activities,\;or\;participants)} \end{array}$$

For resources with costs measured annually, we calculated the daily cost by dividing the annual cost by a fixed value of 245 site-days per year for both facility-adjacent and community-based sites. For resources with variable costs (consumables and Xpert equipment owned and operated outside of the screening intervention), we directly estimated a cost per participant.

We designated equipment into two categories: equipment utilized to support a specific screening step, and equipment required to support the overarching screening process. For equipment utilized for a specific step (e.g., X-ray generators), we calculated the cost per use by dividing the cost per site-day by the observed number of events (e.g., X-rays performed) per day. For equipment utilized to support the overarching process (e.g., tents), we estimated a per-participant cost by dividing the daily equipment cost by the average number of participants screened per day. For equipment designed to last multiple years, we applied straight-line depreciation to equipment costs by dividing the total cost by the useful life and further reduced the practical capacity by a factor of 0.9 to account for potential repairs, calibrations, and maintenance.

We calculated personnel costs by estimating daily salaries according to job type. For community-based screening, we included an average daily cost for physical space that was comprised of venue and local authority payments, and electricity when used instead of generator fuel. For facility-adjacent screening, we utilized space that would otherwise have gone unused; thus, no opportunity costs were assumed to be incurred for space in this arm.

We defined implementation costs as the overhead expenses necessary to support all background activities essential for implementing the intervention. We categorized activities as either one-off activities required at the start of a screening program or ongoing activities required to maintain operations [19]. We developed a narrative outline of all implementation activities and used empirical study data to estimate the annual cost per activity per site. One-off implementation activities required at the start of the screening program, such as staff training, community sensitization, and site preparation, were amortized over the projected implementation period. We excluded costs incurred solely for research purposes, including research data collection, trial-specific monitoring, and research staff activities not required for routine implementation. A full outline of implementation activities and costs, including the frequency, time horizon, and annualized cost assumptions for each implementation activity, is detailed in S2 Appendix.

### Costs per screening step

To estimate the cost of supporting each step in the screening cascade, we first developed a process map for the screening intervention through a combination of observations of staff workflows and discussions with study staff. The process map captures the series of steps a participant would complete from arrival at the screening site to return for follow-up Xpert test results: initial intake, X-ray screening, counseling after X-ray, sputum collection, and delivery of Xpert results.

We calculated the cost per step by calculating all step-specific resource costs and summing them. We used time-and-motion data to assign time units to each component of the screening intervention involving participant-staff interaction, then calculated the proportion of participant time spent on that step on an average day across all participants. For fixed-cost resources not associated with a specific screening step, we assigned costs to each screening step by multiplying the proportion of participant time spent on that step by the estimated cost per participant. For the X-ray, sputum collection, and Xpert testing steps, we then added the step-specific equipment cost per participant. For sputum collection and Xpert testing, we also added the step-specific consumable cost per individual completing that step.


$$\begin{array}{c} Cost\;per\;step=Equipment\;cost+Staff\;cost+Physical\;space\;cost+Implementation\;cost+Consumable\;cost \end{array}$$

$$\begin{array}{c} =\left[\left(\frac{General\;equipment\;cost}{participant}\times \;\%\;overall\;daily\;participant\;time\;on\;step\right)\;+\frac{{Step\;specific\;equipment\;cost}^{a}}{participant}\right] \end{array}$$

$$\begin{array}{c} +\;\left(\frac{Staff\;salary}{minute}\times \frac{minutes}{step}\right) \end{array}$$

$$\begin{array}{c} +\left(\frac{{Space\;cost}^{b}}{minute}\times \frac{minutes}{step}\right) \end{array}$$

$$\begin{array}{c} +\left(\frac{Implementation\;cost}{participant\;}\times \%\;overall\;daily\;participant\;time\;spent\;on\;step\right) \end{array}$$

$+\frac{{Consumable\;cost}^{c}}{participant}$

^a^ Direct equipment costs were added to the x-ray, sputum collection, and TST placement steps.

^b^ Space costs were only included in cost calculations for community-based screening.

^c^ Consumable costs were added to the sputum collection/Xpert testing and TST placement steps.

### Costs per testing activity and per positive result

**Fig 1:**
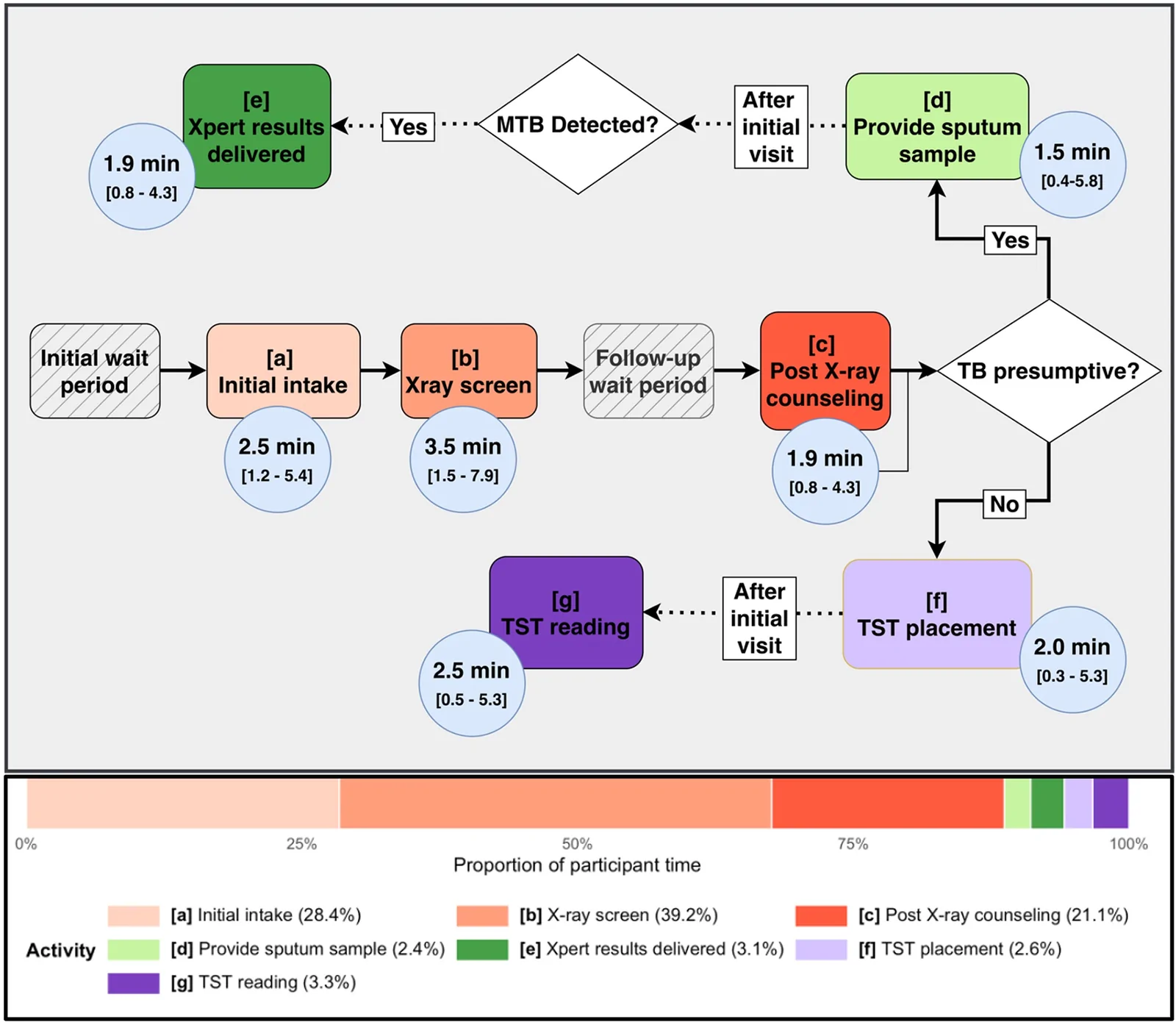
Process map with staff time estimates and proportion of participant time spent per activity within a mobile chest X-ray screening intervention for tuberculosis in Uganda. Top panel: The process map outlines the steps required to complete screening for tuberculosis (TB) and TB infection as part of a community-wide active case-finding intervention in Uganda. Grey boxes indicate participant wait time, and colored rectangular boxes indicate participant-staff interaction during initial or return visits. Diamonds indicate decision points, and dotted lines indicate transitions from the initial to the return visit. Staff time estimates (in minutes, with 95% uncertainty intervals) required for each participant-staff interaction step are provided in blue circles. These staff time estimates only account for time that staff actively spent with participants and do not account for time that participants spent waiting at the screening site or producing sputum samples TST-related workflow steps are shown for completeness because they were observed during the screening workflow, but TST costs are not included in the active TB cost outcomes reported in this analysis. TB = tuberculosis; TST = tuberculin skin test; MTB = mycobacterium tuberculosis. Bottom panel: The proportion of participant time spent on each activity among the full participant population, with data from facility and community sites combined. Steps required for initial x-ray screening (initial intake, x-ray, and counseling) comprised the bulk of participants’ time at both site types, with an average of 45 x-rays conducted daily. On average, 6.5 participants provided sputum for Xpert testing per day, and 5.4 participants participated in TST screening.

To calculate the cost per diagnostic testing activity (i.e., per sputum submitted), we first calculated the cost per individual completing the initial screening steps required of all participants (i.e., intake, X-ray, and follow-up counseling; see Fig 1). We then estimated the number of participants screened per individual submitting a sputum sample for Xpert testing. We multiplied these values by the cost per individual screened to calculate the total screening cost per diagnostic testing activity. We then added the incremental per-participant diagnostic cost for Xpert testing to the screening cost to calculate the total cost (inclusive of both screening and diagnostic testing) per sputum submitted.

To calculate the cost per positive Xpert result returned, we first estimated the number of participants needed to test to produce a positive Xpert result. We then multiplied the number needed to test by the cost per sputum submitted. To estimate the cost per result returned (inclusive of both screening, diagnostic testing, and delivery of results), we then added the additional per-participant cost to deliver Xpert results. The costs to deliver results do not account for transportation or opportunity costs incurred by individual participants to return to the screening site.

We conducted a probabilistic sensitivity analysis (PSA) to propagate uncertainty in the active TB diagnostic cascade and costing model. Cascade probabilities—including the probability of qXR score ≥0.1 among participants with valid qXR scores, sputum submission among participants with qXR score ≥0.1, direct-to-Xpert sputum submission among participants without completed CXR (e.g., pregnant women), valid Xpert results among participants submitting sputum, and Xpert positivity among those with valid Xpert results—were sampled independently from beta distributions using observed numerators and denominators with Jeffreys priors. To reflect uncertainty in cost-model inputs, we empirically bootstrapped daily screening and sputum volumes by resampling observed screening site-days with replacement within site type, varied participant-time inputs using lognormal distributions, and applied a lognormal resource-cost multiplier centered at 1.0 with a coefficient of variation of 0.05 to resource-cost inputs. We recalculated the full costing model across 500 simulations of the cost model and sampled these simulations with replacement across 10,000 simulations of the screening cascade. For each simulation, we estimated the cost per 1,000 participants screened as the cost of CXR screening plus the cost of sputum/Xpert testing, divided by the simulated number of Xpert-positive results. Full model inputs and sampling approach are summarized in S1 Table.

The primary trial is still ongoing, and arm-specific results are masked to investigators; thus, for purposes of this costing analysis, we present costs per diagnostic testing activity and per positive result returned as averaged across community-based and facility-adjacent settings.

### Sensitivity analyses

We conducted sensitivity analyses to assess the extent to which variations in operational factors of programmatic interest affected cost estimates for Xpert testing. Holding all other variables constant, we varied the daily screening volume from 10 to 80 X-rays and analyzed the impact on the cost per positive Xpert. We also used screening data to estimate the impact of increasing the CXR risk threshold for sputum collection on the percentages of participants who submitted sputum and received positive Xpert results, and, subsequently, the cost per positive Xpert. Finally, to assess generalizability across epidemiologic contexts, we varied the number of positive Xpert tests (per 1,000 people screened) from 0.5 to 2.0 times the observed value under two separate assumptions (increase/decrease in proportion of Xpert tests positive and increase/decrease in number of Xpert tests performed) and estimated the resulting cost per Xpert-positive result under each.

## Results

Across 1,915 screening site-days, a mean of 47.3 participants were screened per site-day (SD 22.1; median 47, IQR 32.5–61.0). Among 93,650 age-eligible participants during the analysis period, 90,668 completed CXR. Of these, 14,318 had qXR scores ≥0.1, and 12,954 submitted sputum after a qXR-positive screen; an additional 1,676 participants, primarily those ineligible for CXR because they were pregnant, submitted sputum for Xpert testing without completing CXR. The distribution of qXR scores among participants with valid qXR results is shown in S1 Fig.

Overall, 14,630 sputum specimens were collected for Xpert testing, and 511 were Xpert-positive, corresponding to 5.6 Xpert-positive results per 1,000 participants screened by CXR. A full summary of cascade results with 95% confidence intervals is provided in S2 Table, and a summary of participant characteristics is provided in S3 Table.

The process map summarizing the steps of the screening intervention and their associated time measurements is shown in Fig 1.

**Fig 2:**
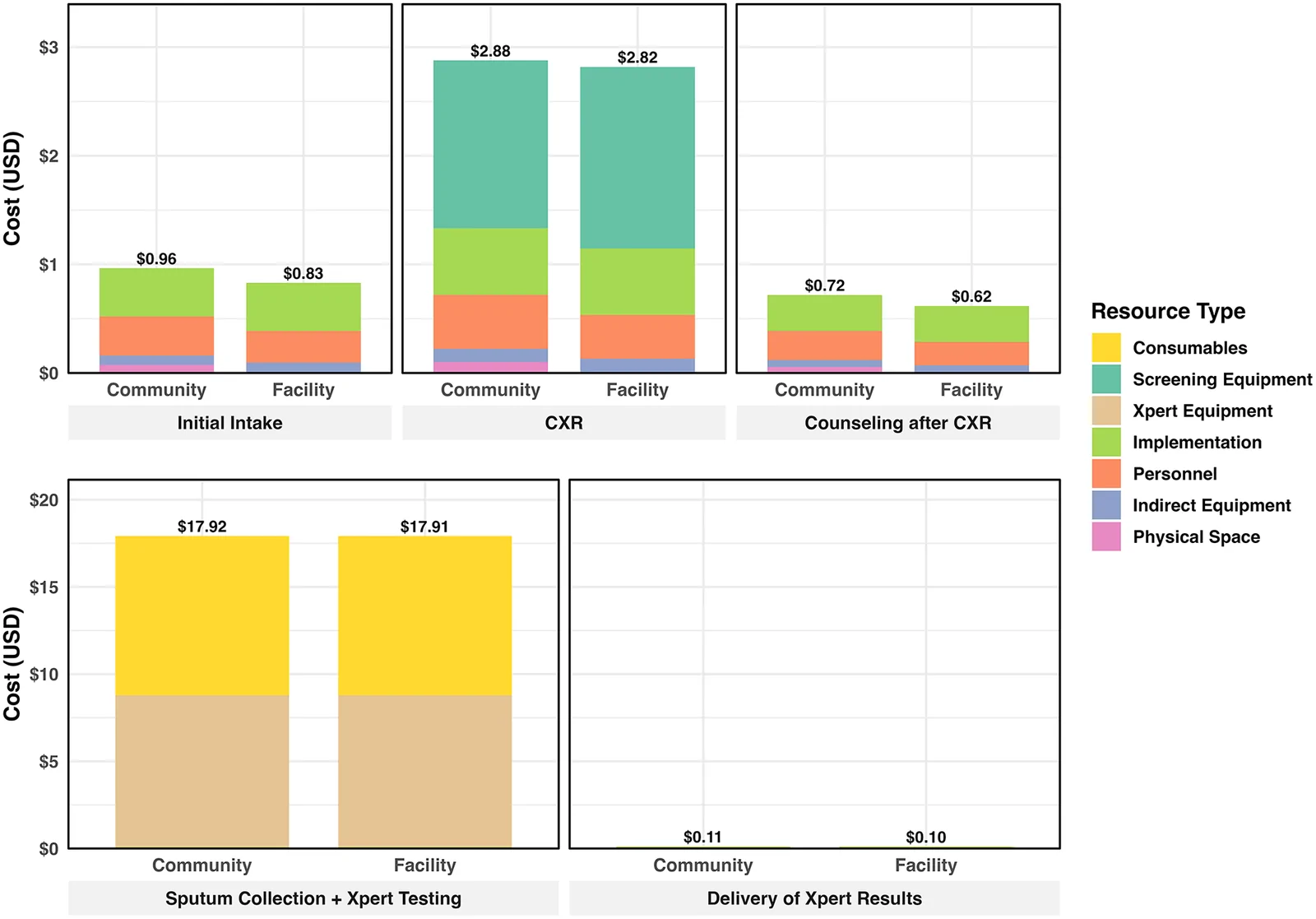
Breakdown of resource costs for community-based and facility-adjacent tuberculosis screening, per activity completed by an individual participant. Moving top to bottom, the panels show the steps required for an individual participant to complete initial CXR screening and Xpert testing. Resource costs are shown per activity completed and use site-specific daily CXR and sputum volumes for community-based and facility-adjacent screening sites. Because final arm-specific trial outcomes remain masked, these per-activity costs do not account for strategy-specific differences in qXR positivity, sputum submission, Xpert positivity, treatment initiation, or downstream effectiveness, and should not be interpreted as a comparison of the overall efficiency of the two trial strategies. CXR: chest X-ray; qXR: computer-aided detection software for chest X-ray interpretation; TB: tuberculosis; Xpert: Xpert MTB/RIF or Xpert MTB/RIF Ultra.

Resource costs were largely similar for community-based and facility-adjacent testing, except that daily staff transportation to community-based sites resulted in higher annual implementation and personnel costs (because community-based screening required a paid driver to join each screening team) and incurred an additional daily fee of $13 per site paid to the local government authority. For the subset of participants who submitted sputum for Xpert testing, Xpert equipment ($8.71 per participant) and cartridges ($9.13 per participant) were the costliest individual items (full resource-specific costs are tabulated in S4 Table). Xpert testing was correspondingly the most expensive individual step (Fig 2, with results also tabulated in S5 Table), followed by X-ray screening.

For participants who completed the initial screening without submitting sputum, the cost per participant screened was $4.41. In our screening population and at the selected CAD score threshold, 6.2 participants completed CXR screening for every sputum specimen submitted for Xpert testing, resulting in a cost of $45 per sputum specimen submitted. Of this amount, approximately $27 reflected initial CXR screening costs incurred across all participants screened, while approximately $18 reflected the incremental costs of sputum collection, Xpert testing, and Xpert result delivery among participants who submitted sputum.

Overall, 26 participants provided sputum samples for each positive Xpert result, resulting in an average cost of $ 1,299 per positive Xpert result (95% uncertainty interval: $1,134-$1,488; Table 1). The total estimated cost of the active TB screening and diagnostic pathway during the analysis period was $664,000, corresponding to $7.32 per participant screened by CXR.

**Table 1:** Activities and costs per positive test for TB disease in a population-based screening trial.

Outcome	Participant Activities per Activity/ Result		Cost per Activity/ Result (95% UI)
*X-rays*	*Sputum Samples*
X-ray Performed	1.0	–	$4.41 ($3.97-$4.88)
Sputum Submitted	6.2	1.0	$45 ($41-$50)
Positive Xpert Result	177	28.6	$1299 ($1,134-$1,488)

### Sensitivity analyses

**Fig 3:**
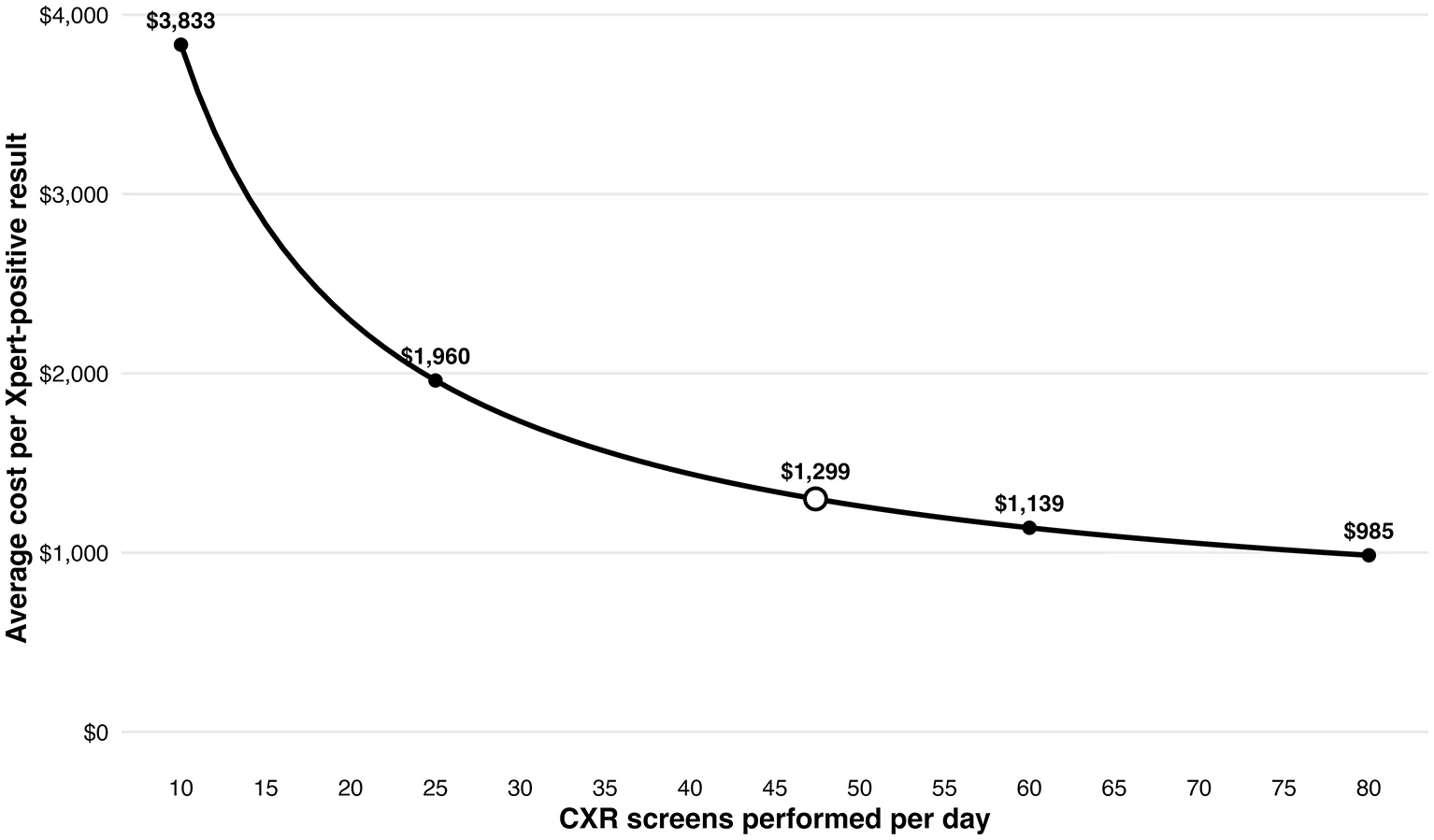
Variation in cost per positive Xpert as a function of daily X-ray volume. Costs per positive Xpert were averaged across facility-adjacent and community-based sites, assuming a daily X-ray volume that varied between 10 and 80 X-rays per day. Our observed daily x-ray volume of 47.3 x-rays per day is represented by the open circle.

Across the full screening sample period, we observed an average of 47.3 X-rays performed per day across all screening sites. If the daily X-ray volume was reduced to ten per day (holding other variables constant), the estimated cost per positive Xpert result would rise to $3,833 (Fig 3). Conversely, increasing the daily X-ray volume to 80 would reduce this cost per positive Xpert to $985.

**Fig 4:**
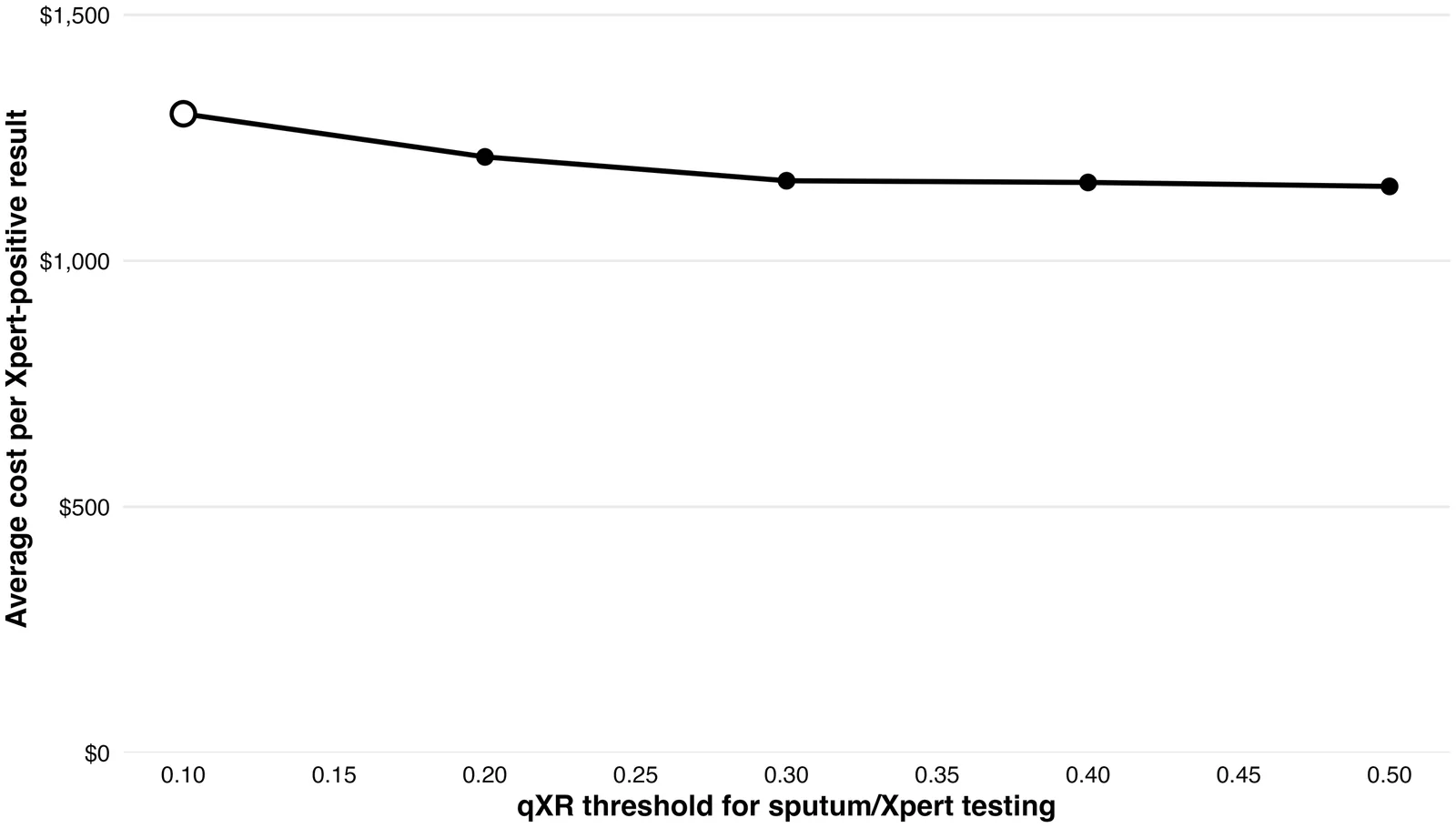
Variation in cost per positive Xpert as a function of CXR risk score threshold. The present study utilized a risk threshold of 0.1 (qXR, qure.ai) as a positive screen, prompting participants to provide a sputum sample for follow-up diagnostic testing via Xpert Ultra. This figure illustrates how the cost per positive Xpert result would vary if that risk threshold were to be made more stringent.

Increasing the stringency of the CXR risk threshold for obtaining sputum (from 0.1 to 0.5) would result in an estimated 6.5% of participants screening positive (down from 16%) and 7.6% of Xpert results returning positive (up from 3.5%), but with only modest reduction in the average cost per positive Xpert test (from $1,299 to $1,151; Fig 4, Table 2). Under the less stringent threshold of 0.1, the cost per positive Xpert result included $783 in CXR screening costs and $516 in Xpert diagnostic costs. Under the more stringent threshold of 0.5, 30 more CXR screens would be required per Xpert-positive result, increasing screening costs by $128, but 15 fewer sputum samples would need to be submitted per Xpert-positive result, saving $276 in diagnostic costs (Table 2). The incremental cost per additional positive Xpert was $1,329 when lowering the CXR threshold for Xpert testing from 0.5 to 0.3, versus $2,816 when further lowering the threshold from 0.3 to 0.1.

**Table 2:** Volume of activities per 1000 people screened and associated costs of X-ray screening and diagnostic testing for TB, per positive Xpert result, varied by X-ray risk threshold.

X-ray risk threshold	Activities per 1000 people screened					Cost per positive Xpert		
	X-rays performed	Sputum samples submitted	Positive Xpert results returned	% of participants submitting sputum	% of sputum specimens testing positive	Screening cost	Diagnostic cost	Total cost
0.1	1000	161	5.6	16.1%	3.5%	$783	$516	$1,299
0.2	1000	112	5.3	11.2%	4.8%	$830	$381	$1,211
0.3	1000	89	5.2	8.9%	5.9%	$853	$310	$1,163
0.4	1000	74	5.0	7.4%	6.7%	$889	$270	$1,159
0.5	1000	65	4.8	6.5%	7.6%	$911	$240	$1,151

**Fig 5:**
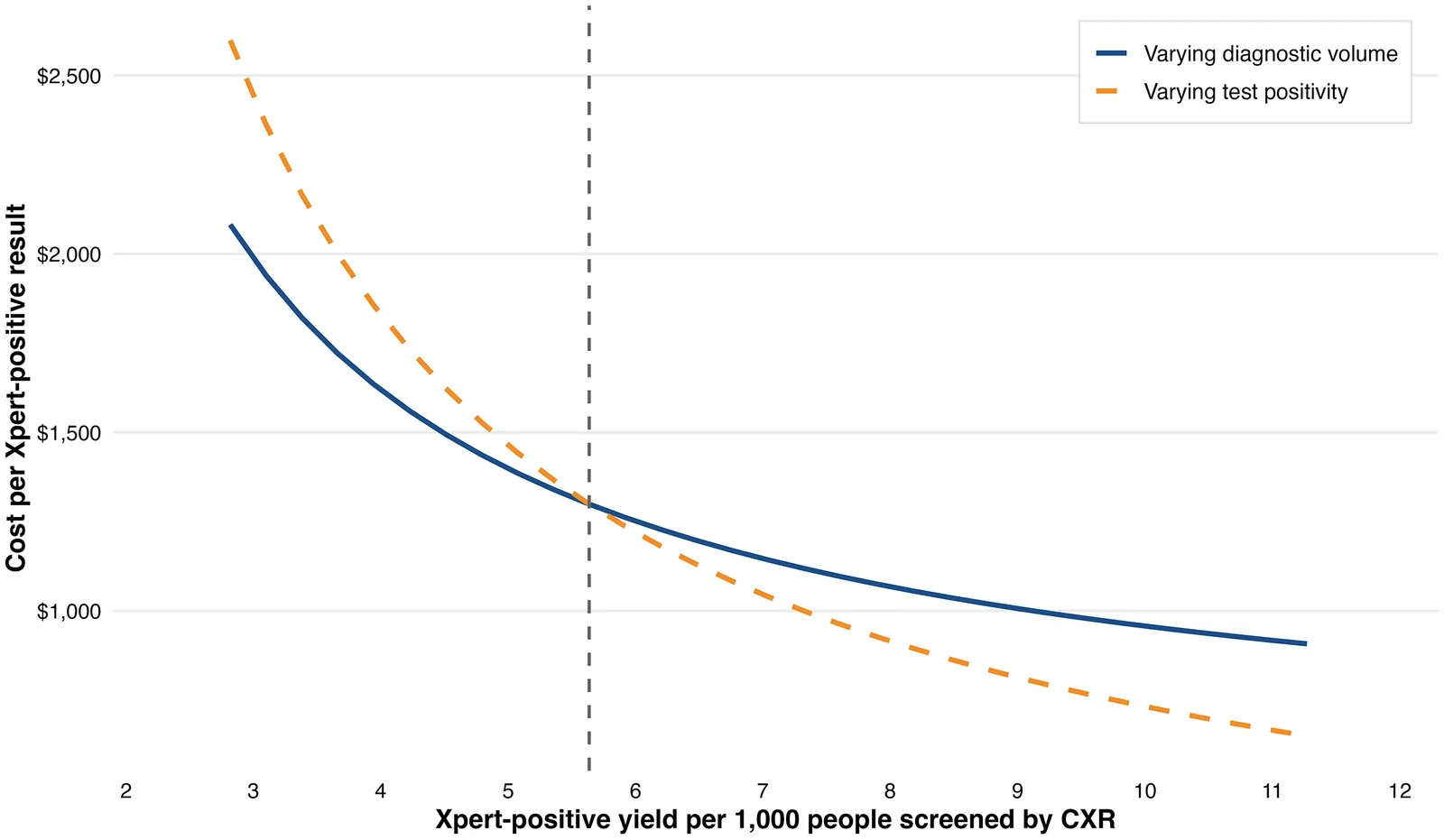
Sensitivity of cost per initial Xpert-positive result to Xpert-positive yield. The estimated cost per initial Xpert-positive result varies across lower- and higher-yield screening scenarios, illustrating the dependence of cost-per-positive estimates on local TB burden and diagnostic yield. The vertical dashed line indicates the observed baseline yield of 5.6 initial Xpert-positive results per 1,000 participants screened by CXR. The dashed orange line shows how the Xpert-positive yield varies while holding screening and diagnostic volumes at observed levels. The solid blue line varies Xpert-positive yield while allowing diagnostic volume to scale with yield. CXR: chest X-ray; TB: tuberculosis; Xpert: Xpert MTB/RIF or Xpert MTB/RIF Ultra.

Halving the observed Xpert positivity increased the estimated cost per Xpert-positive result to $2,598, while doubling it reduced the cost to $650. Doubling and halving the number of Xpert tests performed (keeping positivity levels constant) resulted in cost-per-positive-Xpert estimates of $2,082 and $908, respectively (Fig 5).

## Discussion

We evaluated the costs of implementing mobile chest X-ray screening for tuberculosis in communities and health facilities across peri-urban Uganda. The estimated cost per positive Xpert result was $1299. Community-based strategies for detecting TB disease are costly, but the cost per positive result varies substantially with parameters that program planners could feasibly target to optimize future investments.

These results provide a framework for making operational and budgetary decisions when planning the programmatic implementation of a similar intervention. For example, if a TB program aimed to deploy a single implementation team to diagnose an additional 100 people with TB in a population with a similar TB risk to that of this study, that team would need to screen approximately 18,200 individuals and submit close to 2600 sputum samples for Xpert testing, resulting in a total cost of $128,100.

While the CXR risk threshold plays an important role in the total cost of screening, and the threshold of 0.1 used in the present study is lower than recommended thresholds in programmatic contexts, we found that making this threshold more stringent only modestly reduced the average cost per positive Xpert result. This finding reflects an increased cost per positive Xpert result from X-ray screening when the Xpert testing threshold is higher, which partially offsets the lower cost of diagnostic testing. As examples, lowering the CXR threshold from 0.5 to 0.3 would increase the number of Xpert-positive TB diagnoses by about 6%, at the same cost per positive result. Further lowering the threshold from 0.3 to 0.1 would increase the number of Xpert-positive diagnoses by an additional 10%, but at an incremental cost of $2816 per additional positive diagnosis. This additional incremental cost may still be justified, depending on the resources available and the cost-effectiveness threshold of the program. Future work should integrate cost analyses like this one with diagnostic accuracy assessments, enabling decision-makers to use the CXR risk score threshold as a practical lever for optimizing both efficiency and case detection.

Daily x-ray volume and underlying TB prevalence are important determinants of cost. We observed that daily x-ray volume has a non-linear relationship with cost per person screened, with marginal gains in efficiency above a volume of 30–50 people screened per day (the volume at which diagnostic testing rather than fixed operating expenses becomes the main driver of cost per participant). For example, increasing daily x-rays at community sites from 15 to 20 would reduce the cost of screening per positive Xpert by $515, whereas increasing from 45 to 50 would yield only a $84 reduction. To maximize operational efficiency, X-ray screening programs would need to ensure that enough daily X-rays can be performed over time. Further, because the cost per Xpert-positive result is approximately inversely proportional to yield, implementation in higher-burden settings or subpopulations would reduce the cost per positive result, while lower-yield populations would increase it, independent of operational costs.

In evaluating the cost-effectiveness of TB screening programs like this one, an important consideration is that—under programmatic conditions in some settings—many individuals initiating TB treatment do so without bacteriological confirmation [20]. Some of these people likely have underlying TB, but many also likely represent false-positive results [21]. In settings where clinical diagnosis based on CXR screening is common, the cost per positive Xpert result may not be the most appropriate measure of cost-effectiveness, and cost-effectiveness is likely to depend strongly on the proportion of clinical diagnoses that represent true underlying TB. While this study did not include these unconfirmed diagnoses in the primary trial outcome, future studies should consider their importance to the cost-effectiveness of TB screening programs.

Our approach was strengthened by the use of prospective observational data. By estimating and assigning costs to individual participant activities, this detailed micro-costing approach enhanced our ability to assess the sensitivity of our cost estimates to variation in key operational factors such as daily x-ray volume and qXR threshold.

Our costing approach also had some notable limitations. The research context may limit generalizability to routine programmatic settings, where implementation costs could differ. Activities conducted specifically for this standalone program—such as staff training, community sensitization, and site visits—might be integrated into broader NTP operations, reducing marginal costs. We included one-time start-up costs in the costing model but did not separately estimate a temporary start-up period with reduced efficiency and screening volume. Programs newly implementing mobile CXR screening may experience lower short-term efficiency if fixed costs are incurred before screening volume stabilizes.

Time estimates from our process map may also vary under real-world conditions, where staff qualifications, motivation, and availability are less controlled. Due to capacity limitations, we excluded the costs of certain follow-up activities that occurred outside of regular business hours and/or the defined study sites. To the extent that such activities would be required to follow up participants with positive results, our measured costs may underestimate the true cost of a comprehensive screening activity.

Our cost estimates are also context-specific and may not generalize to settings with substantially different socioeconomic conditions. However, our findings regarding the relative influence of different parameters on the cost per positive result are likely transportable across many settings. To maintain blinding during the ongoing trial, we combined prevalence estimates across strategies. While our estimates are useful for budget impact analyses, full cost-effectiveness evaluations will require comparison with corresponding strategy-specific effectiveness data. Future efforts should compare the cost of TB screening across multiple settings, using different screening algorithms and a range of TB prevalence levels.

## Conclusion

We estimated the costs of implementing mobile chest x-ray screening for TB disease in a peri-urban Ugandan setting. We found that while the cost per positive Xpert result was high, it could be meaningfully reduced by optimizing modifiable program design factors. By quantifying costs and identifying opportunities to improve efficiency, our results provide evidence-based economic benchmarks for TB programs seeking to invest in mobile CXR for community- or facility-based active case finding in high-burden settings such as Uganda.

## Supporting information

S1 Appendix Capacity cost rate equations by resource group. Capacity cost rates (i.e., the unit cost as a function of practical capacity) were calculated distinctly for each resource group: equipment, personnel, physical space, and implementation activities.
(DOCX)

S2 Appendix Implementation activities required to support the intervention and associated costs. (DOCX)

S1 Table Inputs and sampling approach for the probabilistic sensitivity analysis. (DOCX)

S2 Table Active tuberculosis diagnostic cascade among age-eligible participants screened by mobile chest X-ray. (DOCX)

S3 Table Characteristics of age-eligible participants completing CXR screening. (DOCX)

S4 Table Unit costs for each resource category per site and the associated capacity cost rates (CCRs) for each screening site type. (DOCX)

S5 Table Total resource costs per screening step, separated by individual resource group and stratified by facility-adjacent and community-based screening. (DOCX)

S1 Fig Distribution of qXR TB risk scores among participants with valid qXR results. Bars show the percentage of participants in each qXR score band; labels show the number and percentage of participants in each band. Score bands are shown in 0.1 increments, with additional bands above 0.5 included to display the upper tail of the qXR distribution. qXR: computer-aided detection software for chest X-ray interpretation; TB: tuberculosis.
(TIFF)
