# Supplementary material for: Resources to implement general population mobile chest x-ray screening for tuberculosis in Uganda: A trial-based costing analysis
Source: PLOS Glob Public Health. 2026 Sep 10;6(9):e0005950. doi: 10.1371/journal.pgph.0005950 (PMC13561341; doi:10.1371/journal.pgph.0005950)
Supplement: Supplementary file 1 [file pgph.0005950.s001.docx]

**S1 Appendix.** Capacity cost rate equations by resource group.

**Equation 1:** Calculation of the capacity cost rate for equipment utilized for a specific screening step, using the x-ray step as an illustrative example.

$$CCR= \frac{Resource cost}{Practical capacity}$$

$$=\frac{\frac{Annual xray equipment cost}{Annual site days}}{\frac{Xrays conducted}{Day}}$$

$$=\frac{\$}{day}\times\frac{day}{xray}$$

$$=\frac{\$}{xray}$$

**Equation 2:** Calculation of the capacity cost rate for equipment supporting all screening steps.

$$CCR= \frac{Resource cost}{Practical capacity}$$

$$=\frac{\frac{Annual equipment costs per site}{Annual site days}}{\frac{Screening participants}{Day}}$$

$$=\frac{\$}{day}\times\frac{day}{screening participants}$$

$$=\frac{\$}{screening participant}$$

**Equation 3:** Calculation of the capacity cost rate for personnel.

$$CCR= \frac{Resource cost}{Practical capacity}$$

$$=\frac{\frac{Total daily staff salaries}{Day}}{\frac{Screening participants}{Day}}$$

$$=\frac{\$}{day}\times\frac{day}{screening participants}$$

$$=\frac{\$}{screening participant}$$

**Equation 4:** Calculation of the capacity cost rate for physical space.

$$CCR= \frac{Resource cost}{Practical capacity}$$

$$=\frac{\frac{Site fee}{Day}}{\frac{Screening participants}{Day}}$$

$$=\frac{\$}{day}\times\frac{day}{screening participants}$$

$$=\frac{\$}{screening participant}$$

**Equation 5:** Calculation of the capacity cost rate for implementation costs.

$$CCR= \frac{Resource cost}{Practical capacity}$$

$$=\frac{\frac{Annual implementation costs per site}{Annual site days}}{\frac{Screening participants}{Day}}$$

$$=\frac{\$}{day}\times\frac{day}{screening participants}$$

$$=\frac{\$}{screening participant}$$
