# Supplementary material for: Resources to implement general population mobile chest x-ray screening for tuberculosis in Uganda: A trial-based costing analysis
Source: PLOS Glob Public Health. 2026 Sep 10;6(9):e0005950. doi: 10.1371/journal.pgph.0005950 (PMC13561341; doi:10.1371/journal.pgph.0005950)
Supplement: Supplementary file 2 [file pgph.0005950.s002.docx]

**S2 Appendix. Implementation activities required to support the intervention and associated costs.**

| **One-time costs (per 5-year period, scaled to annual costs for calculations)** | | | |
| --- | --- | --- | --- |
| **Item/activity** | **Cost** | **Unit** | **Assumptions** |
| Shipping cost | $1,368.75 | Per x-ray machine |  |
| Customs clearance cost | $405.41 | Per x-ray machine |  |
| AEC/Radiation safety cost | $1,118.92 | Per x-ray machine |  |
| Shipping cost | $108.00 | Per CAD offline box |  |
| Customs clearance cost | $270.27 | Per CAD offline box |  |
| Develop SOPs for X-ray administration, hardware/software troubleshooting and QA/ QC manuals, and management of screening/testing equipment. | $37.50 | per program (all sites) | Calculated as the cost for 3 person-hours of work |
| Recruit staff to administer intervention | $600.00 | per program (all sites) | Calculated as the cost for 48 person-hours of work |
| Sensitization meetings at district health offices and referral health facilities - **staff salaries** | $234.00 | per program (all sites) |  |
| Sensitization meetings at district health offices and referral health facilities - **fuel** | $85.00 | per site |  |
| Sensitization meetings at district health offices and referral health facilities - **transport reimbursements** | $1,080.00 | per program (all sites) |  |
| Initial staff training - **participant per diem** | $8.00 | per program (all sites) | Estimated 4 staff per site |
| Initial staff training - **trainer salaries** | $62.50 | per training | Estimate of a staff member delivering 5 hours of didactic training at $12.5 per hour |
| **Ongoing maintenance costs (scaled to annual totals for calculations)** | | | |
| **Item/activity** | **Cost** | **Unit** | **Assumptions** |
| Conduct sensitization meetings with community leaders and routine clinic staff - staff salaries | $234.00 | per site | Assuming 3 meetings per year |
| Conduct sensitization meetings with community leaders and routine clinic staff - **fuel** | $80.00 | per site | Assuming 3 meetings per year |
| Conduct sensitization meetings with community leaders and routine clinic staff - **transport reimbursements** | $647.50 | per site | Assuming 3 meetings per year |
| Distribute stock from central location to screening sites | $40.00 | per site | Assuming 3 times per year |
| Advertising costs (signs, local radio adverts) | $108.10 | per zone | Assuming 3 times per year |
| Meet individually with very-local leaders at community screening sites - **staff salaries** | $104.00 | per meeting | Assuming 10 meetings per site, 3 times per year |
| Meet individually with very-local leaders at community screening sites - **fuel** | $50.00 | per study zone | Assuming 10 meetings per site, 3 times per year |
| Computer IT/hardware support | $2,433.60 | per program (all sites) |  |
| Transport of staff (and supplies) between central hub (Kampala) and screening regions (other districts) | $40.00 | per COMMUNITY site | Biweekly cost |
| Daily transport from lodging to screening sites, for hotspot intervention | $3.00 | per COMMUNITY site | Biweekly cost |
| Storage of materials | $108.10 | per site | Monthly cost |
| Provide refresher trainings for staff | $62.50 | per training | Once yearly |
| Staff per diem (site teams) | $5.50 | per staff | Daily cost |
| Housing support costs (site teams) | $135.10 | per site | Monthly cost |
| Internet/mobile phone minutes (site teams) | $27.00 | per staff | Monthly cost |
| Consultancy fee for RSO | $432.40 | per site | 4 times a year |
| Supervisory site visits from management team - **staff salaries** | $162.50 | per visit | 3 times a month |
| Supervisory site visits from management team - **fuel** | $55.00 | per visit | 3 times a month |
