# Supplementary material for: Resources to implement general population mobile chest x-ray screening for tuberculosis in Uganda: A trial-based costing analysis
Source: PLOS Glob Public Health. 2026 Sep 10;6(9):e0005950. doi: 10.1371/journal.pgph.0005950 (PMC13561341; doi:10.1371/journal.pgph.0005950)
Supplement: Supplementary file 3 [file pgph.0005950.s003.docx]

**S1 Table.** Inputs and sampling approach for probabilistic sensitivity analysis.

| **Model component** | **Parameter varied** | **Central value / observed input** | **Sampling approach** | **Role in model** |
| --- | --- | --- | --- | --- |
| Active TB cascade | qXR score ≥0.1 among participants with valid qXR scores | 14,318 / 90,649 = 15.8% | Beta distribution with Jeffreys prior: Beta(14,318 + 0.5, 90,649 - 14,318 + 0.5) | Determines the number of participants entering the qXR-driven sputum/Xpert pathway |
| Active TB cascade | Sputum submission among participants with qXR score ≥0.1 | 12,954 / 14,318 = 90.5% | Beta distribution with Jeffreys prior: Beta(12,954 + 0.5, 14,318 - 12,954 + 0.5) | Determines qXR-pathway sputum/Xpert volume |
| Active TB cascade | Direct-to-Xpert sputum submission without completed CXR, modeled per CXR completed | 1,676 / 90,668 = 1.8% | Beta distribution with Jeffreys prior: Beta(1,676 + 0.5, 90,668 - 1,676 + 0.5) | Adds sputum/Xpert volume generated by participants who did not complete CXR |
| Active TB cascade | Valid initial Xpert result among all sputum specimens in the Xpert costing pathway | 14,502 / 14,630 = 99.1% | Beta distribution with Jeffreys prior: Beta(14,502 + 0.5, 14,630 - 14,502 + 0.5) | Determines the number of valid initial Xpert results |
| Active TB cascade | Initial Xpert-positive result among participants with valid initial Xpert results | 511 / 14,502 = 3.5% | Beta distribution with Jeffreys prior: Beta(511 + 0.5, 14,502 - 511 + 0.5) | Determines Xpert-positive yield per 1,000 participants screened |
| Operational volume | Daily CXR volume, community-based/hotspot sites | Mean 49.3 participants per cluster-day | Empirical bootstrap from observed cluster screening-days | Affects fixed-cost spreading and cost per CXR screen |
| Operational volume | Daily CXR volume, facility-adjacent sites | Mean 45.5 participants per cluster-day | Empirical bootstrap from observed cluster screening-days | Affects fixed-cost spreading and cost per CXR screen |
| Operational volume | Daily sputum volume, community-based/hotspot sites | Median 7.66 sputum submissions per cluster-day in cost-model draws | Empirical bootstrap from observed cluster screening-days | Affects activity-time allocation and sputum/Xpert cost |
| Operational volume | Daily sputum volume, facility-adjacent sites | Median 7.57 sputum submissions per cluster-day in cost-model draws | Empirical bootstrap from observed cluster screening-days | Affects activity-time allocation and sputum/Xpert cost |
| Resource costs | Overall resource-cost multiplier | Centered at 1.00 | Lognormal distribution with CV = 0.05 | Varies resource costs while preserving positive cost values |
| Participant-time inputs | Activity-duration inputs used in TDABC allocation | Initial intake 2.54 min; CXR 3.51 min; post-CXR counseling 1.89 min; sputum 1.47 min; Xpert results 1.89 min; TST placement 1.96 min; TST reading 2.49 min | Lognormal variation around central estimates with CV = 0.15 | Varies activity-time shares used to allocate personnel, implementation, indirect, and space costs across activities |
| Cost-model output | Cost per CXR screen | Deterministic value $4.41 | Recalculated in each cost-model draw | Applied to all participants screened by CXR |
| Cost-model output | Incremental sputum/Xpert diagnostic bundle cost | Deterministic value $18.02 | Recalculated in each cost-model draw | Applied to sputum/Xpert volume from qXR-driven and direct-to-Xpert pathways |
| Final PSA outcome | Cost per Xpert-positive result | Deterministic value $1,299 | Calculated for each PSA draw as cost per 1,000 screened divided by Xpert-positive results per 1,000 screened | Main uncertainty outcome |

Parameters varied in the probabilistic sensitivity analysis, the central values used in the deterministic model, and the sampling approach used to propagate uncertainty. The PSA used 10,000 cascade draws and 500 full cost-model draws; cost-model draws were sampled with replacement across the full set of cascade PSA draws. The analysis included both qXR-driven sputum testing and direct-to-Xpert sputum testing among participants who submitted sputum without completed CXR.

CXR: chest X-ray; qXR: computer-aided detection software for chest X-ray interpretation; PSA: probabilistic sensitivity analysis; TDABC: time-driven activity-based costing; Xpert: Xpert MTB/RIF or Xpert MTB/RIF Ultra; CV: coefficient of variation.
