# Supplementary material for: Resources to implement general population mobile chest x-ray screening for tuberculosis in Uganda: A trial-based costing analysis
Source: PLOS Glob Public Health. 2026 Sep 10;6(9):e0005950. doi: 10.1371/journal.pgph.0005950 (PMC13561341; doi:10.1371/journal.pgph.0005950)
Supplement: Supplementary file 4 [file pgph.0005950.s004.docx]

**S2 Table.** Active tuberculosis diagnostic cascade among age-eligible participants screened by mobile chest X-ray.

| **Screening step** | **Numerator** | **Denominator** | **Denominator Description** | **Percent completing step** | **95% CI (low)** | **95% CI (high)** |
| --- | --- | --- | --- | --- | --- | --- |
| Consented/enrolled | 91417 | 93650 | Age-eligible records in analysis period | 97.6% | 97.5% | 97.7% |
| CXR completed | 90668 | 91417 | Consented/enrolled | 99.2% | 99.1% | 99.2% |
| Valid qXR score | 90649 | 90668 | CXR completed | 100.0% | 100.0% | 100.0% |
| qXR >= 0.1 / presumptive TB | 14318 | 90649 | Valid qXR score | 15.8% | 15.6% | 16.0% |
| Sputum collected after qXR-positive screen | 12954 | 14318 | qXR >= 0.1 | 90.5% | 90.0% | 90.9% |
| Sputum collected without completed CXR | 1676 | 91417 | Consented/enrolled | 1.8% | 1.7% | 1.9% |
| Total sputum collected for Xpert testing | 14630 | 91417 | Consented/enrolled | 16.0% | 15.8% | 16.2% |
| Valid Xpert result available | 14502 | 14630 | Total sputum collected for Xpert testing | 99.1% | 99.0% | 99.3% |
| Xpert-positive result | 511 | 14502 | Valid Xpert result available | 3.5% | 3.2% | 3.8% |

The active TB diagnostic cascade during the analysis period, including the number and proportion of participants completing each step and exact binomial 95% confidence intervals. In addition to participants who submitted sputum after a qXR-positive screen, the cascade includes participants who submitted sputum for Xpert testing without completing CXR, reflecting a direct-to-Xpert pathway for participants who could not undergo radiography (e.g., pregnant women). Percentages are calculated using the denominator listed for each step.

CXR: chest X-ray; qXR: computer-aided detection software for chest X-ray interpretation; TB: tuberculosis; Xpert: Xpert MTB/RIF Ultra; CI: confidence interval.
