# Supplementary material for: Resources to implement general population mobile chest x-ray screening for tuberculosis in Uganda: A trial-based costing analysis
Source: PLOS Glob Public Health. 2026 Sep 10;6(9):e0005950. doi: 10.1371/journal.pgph.0005950 (PMC13561341; doi:10.1371/journal.pgph.0005950)
Supplement: Supplementary file 5 [file pgph.0005950.s005.docx]

**S3 Table.** Screening Participant Characteristics

| **Variable** | **Category** | **N** | **Percent**  **(out of 90,668)** |
| --- | --- | --- | --- |
| Screening strategy | Community-based/hotspot | 45863 | 50.6% |
|  | Facility-adjacent | 44805 | 49.4% |
| Sex/gender | Female | 51990 | 57.3% |
|  | Male | 38675 | 42.7% |
|  | Unknown | 3 | 0.0% |
| Age group | 15-24 | 20250 | 22.3% |
|  | 25-34 | 19937 | 22.0% |
|  | 35-44 | 17839 | 19.7% |
|  | 45-54 | 15719 | 17.3% |
|  | 55-64 | 9314 | 10.3% |
|  | 65+ | 7609 | 8.4% |
| Self-reported HIV status | Negative/recent negative test | 45985 | 50.7% |
|  | Positive | 6058 | 6.7% |
|  | Prefer not to disclose | 63 | 0.1% |
|  | Unknown/no recent test | 38561 | 42.5% |
| TB symptoms in previous 30 days | Cough | 23384 | 25.8% |
|  | Sputum | 16799 | 18.5% |
|  | Chest pain | 22898 | 25.3% |
|  | Fever | 14477 | 16.0% |
|  | Drenching night sweats | 2015 | 2.2% |
|  | Loss of body weight | 1002 | 1.1% |
|  | Feeling sick or weak | 8161 | 9.0% |
|  | Coughing up blood | 253 | 0.3% |
|  | Other symptom | 506 | 0.6% |
|  | No symptoms reported | 48680 | 53.7% |
| Previous TB diagnosis | Yes | 1238 | 1.4% |
| Household TB contact | Yes | 971 | 1.1% |
