# Supplementary material for: Resources to implement general population mobile chest x-ray screening for tuberculosis in Uganda: A trial-based costing analysis
Source: PLOS Glob Public Health. 2026 Sep 10;6(9):e0005950. doi: 10.1371/journal.pgph.0005950 (PMC13561341; doi:10.1371/journal.pgph.0005950)
Supplement: Supplementary file 6 [file pgph.0005950.s006.docx]

**S4 Table. Unit costs for each resource category per site and the associated capacity cost rates (CCRs) for each screening site type.**

| Resource Category | Cost Frequency | Cost per site | CCR (Facility) | CCR (Community) |
| --- | --- | --- | --- | --- |
| *Equipment* | | |  |  |
| Xray^1^ | Per year | $18,643 | $1.79 per **x-ray** | $1.61 per **x-ray** |
| Sputum/Xpert | Per year | N/A^2^ | $8.71 per **sample** | $8.71 per **sample** |
| TST | Per year | $28.96^3^ | $0.03 per **TST** | $0.02 per **TST** |
| Other^4^ | Per year | $3,700 | $0.43 per **participant** | $0.32 per **participant** |
| *Personnel* | | |  |  |
| Facility | Per day | $46.69 | $1.10 per **participant** |  |
| Community | Per day | $63.13 |  | $1.33 per **participant** |
| Physical Space | Per day | $13.00 |  | $0.27 per **participant** |
| *Implementation^5^* | | |  |  |
| Facility | Per year | $17,372 | $1.69 per **participant** |  |
| Community | Per year | $19,075 |  | $1.64 per **participant** |
| *Consumables* | | |  |  |
| Sputum/Xpert | Per participant | $9.13 |  |  |
| TST | Per participant | $1.89 |  |  |

^1^X-ray equipment includes mobile x-ray machine and computer-aided detection software and license.

^2^The intervention utilized Gene Xpert Ultra machines at the health facility that were not specific to the intervention. Therefore, we applied a fixed cost per sample ($8.71) that was derived from a study that evaluated the cost of Xpert testing in various contexts throughout Uganda.

^3^TST equipment included one TST cooler box per site. Syringes and vials of tuberculin were classified as medical consumables.

^4^Equipment that was non-specific to one of the screening steps was specified as “other.” This included resources such as tables, chairs, tents, vehicles, tablets, and laptops, among others.

^5^While the annual implementation cost at facility sites was lower than community sites, the cost per participant was higher due to the smaller number of total annual screening days compared to the community sites. A full list of implementation costs is included in **S2 Appendix.**
