# Supplementary material for: Resources to implement general population mobile chest x-ray screening for tuberculosis in Uganda: A trial-based costing analysis
Source: PLOS Glob Public Health. 2026 Sep 10;6(9):e0005950. doi: 10.1371/journal.pgph.0005950 (PMC13561341; doi:10.1371/journal.pgph.0005950)
Supplement: Supplementary file 7 [file pgph.0005950.s007.docx]

**S5 Table. Total resource costs per screening step, separated by individual resource group and stratified by facility-adjacent and community-based screening.**

| Screening Step | Site Type | Equipment | Personnel | Physical Space | Implementation | Consumables | TOTAL |
| --- | --- | --- | --- | --- | --- | --- | --- |
| Initial Intake | **F** | $0.10 | $0.31 | $0.00 | $0.48 | $0.00 | **$0.89** |
|  | **C** | $0.09 | $0.37 | $0.08 | $0.46 | $0.00 | **$1.00** |
| X-ray | **F** | $1.93 | $0.43 | $0.00 | $0.66 | $0.00 | **$3.03** |
|  | **C** | $1.73 | $0.52 | $0.11 | $0.64 | $0.00 | **$2.99** |
| Counseling after X-ray | **F** | $0.08 | $0.23 | $0.00 | $0.36 | $0.00 | **$0.67** |
|  | **C** | $0.07 | $0.28 | $0.06 | $0.34 | $0.00 | **$0.75** |
| Sputum + Xpert Testing | **F** | $8.71 | $0.03 | $0.00 | $0.04 | $9.13 | **$17.91** |
|  | **C** | $8.71 | $0.03 | $0.01 | $0.04 | $9.13 | **$17.92** |
| Return for Xpert Results | **F** | $0.01 | $0.02 | $0.00 | $0.04 | $0.00 | **$0.07** |
|  | **C** | $0.01 | $0.04 | $0.01 | $0.05 | $0.00 | **$0.10** |
| TST Placement | **F** | $0.04 | $0.03 | $0.00 | $0.05 | $1.89 | **$2.01** |
|  | **C** | $0.03 | $0.04 | $0.01 | $0.05 | $1.89 | **$2.02** |
| TST Reading | **F** | $0.01 | $0.03 | $0.00 | $0.05 | $0.00 | **$0.09** |
|  | **C** | $0.01 | $0.05 | $0.01 | $0.06 | $0.00 | **$0.13** |

F = Facility-adjacent sites; C = Community-based sites.
